# Supplementary material for: Mpox Vaccination Hesitancy and Its Associated Factors among Men Who Have Sex with Men in China: A National Observational Study
Source: Vaccines (Basel). 2023 Aug 30;11(9):1432. doi: 10.3390/vaccines11091432 (PMC10534529; doi:10.3390/vaccines11091432)
Supplement: Supplementary file 1 [file vaccines-11-01432-s001.zip › vaccines-2589314-supplementary.pdf]

Supplementary Materials

This supplementary materials formed part of the original submission.

Figure S1. The distribution of 27 MSM social organizations from 21 provinces, municipalities, and autonomous regions of China. 2

Table S1. The number of MSM social organizations for 21 provinces, municipalities, and autonomous regions of China. 3

Table S2. The survey questionnaire. 4

Table S3. Population characteristics by self-funded mpox vaccination hesitation among men who have sex with men in China. 5

Table S4. Influencing factors of self-funded mpox vaccination hesitation among men who have sex with men in China. 6

Table S5. Influencing factors of mpox vaccination hesitation among men who have sex with men living without HIV in China. 7

Table S6. Influencing factors of mpox vaccination hesitation among men who have sex with men living with HIV in China. 8

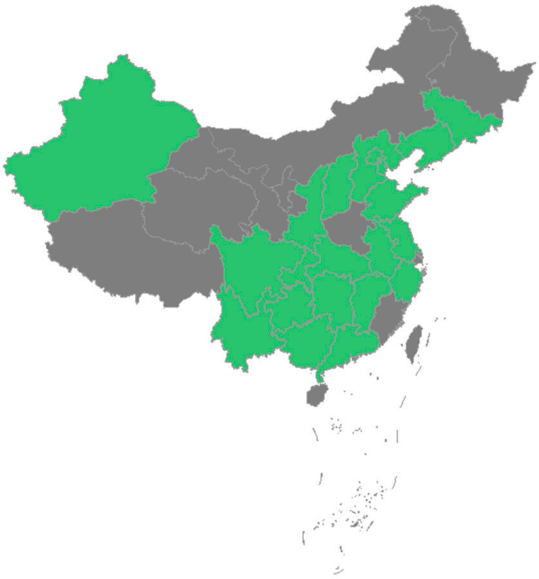

Figure S1. The distribution of 27 MSM social organizations from 21 provinces, municipalities, and autonomous regions of China.
The green area represented that the provinces, municipalities, and autonomous regions were investigated.

Table S1. The number of MSM social organizations for 21 provinces, municipalities, and autonomous regions of China.

Provinces	N
Zhejiang	1
Yunnan	1
Xinjiang	1
Tianjin	1
Sichuan	1
Shanxi	1
Shandong	1
Shaanxi	1
Liaoning	1
Jilin	1
Jiangxi	1
Jiangsu	1
Hunan	2
Hubei	1
Hebei	1
Guizhou	4
Guangxi	1
Guangdong	3
Chongqing	1
Beijing	1
Anhui	1

Table S2. The survey questionnaire.

Variables	Questions
Mpox vaccination hesitancy	If mpox vaccine is available in China, are you willing to get vaccinated?
Age	The period from "Date of birth" to "Survey date"
Occupation	Which followings are your current occupation?
Ethnicity	Which followings are your ethnicity?
Educational level	Which followings are your educational level?
Residence	Which followings are your current residence?
Marital status	Which followings are your marital status?
Sexual orientation	What is your sexual orientation?
Sexual diseases scores	Are you infected with human immunodeficiency virus (HIV)?<br>Are you infected with hepatitis C?<br>Are you currently suffering from sexually transmitted diseases (including syphilis, gonorrhea, condyloma acuminatum, genital herpes and genital chlamydia trachomatis, etc)
Sexual risky behavior	How often have you used condoms during anal sex with men in the last 1 month?<br>Have you had commercial sex with men in the last month?<br>Have you had sex with multiple men in the last month?
Mpox virus infection	After informed participants about the standard, they were asked "Which of the following is your case?"
Mpox-related knowledge	What are the source of mpox infection?<br>What are the possible ways of transmission route of mpox?<br>Who are susceptible to monkeypox?<br>What are the clinical manifestations of mpox?<br>After infected with mpox virus, is there a specific treatment drug?<br>What are measures to avoid getting monkeypox?<br>Is there a mpox vaccine available in China?

Table S3. Population characteristics by self-funded mpox vaccination hesitation among men who have sex with men in China.

Characteristics	Overall (n, %)	Vaccination acceptance (n, %)	Vaccination hesitation (n, %)	P-value
	7538	4046 (53.67)	3492 (46.33)	
**Age group (years)**				<0.001
18-35	5463 (72.47)	3035 (55.56)	2428 (44.44)	
36-59	1941 (25.75)	965 (49.72)	976 (50.28)	
≥60	134 (1.78)	46 (34.33)	88 (65.67)	
**Occupation**				<0.001
Employed	6744 (89.47)	3711 (55.03)	3033 (44.97)	
Unemployed	794 (10.53)	335 (42.19)	459 (57.81)	
**Education level**				<0.001
Junior high school and below	664 (8.81)	301 (45.33)	363 (54.67)	
High school	2990 (39.67)	1505 (50.33)	1485 (49.67)	
Undergraduate	3126 (41.47)	1774 (56.75)	1352 (43.25)	
Postgraduate and above	758 (10.06)	466 (61.48)	292 (38.52)	
**Marital status**				<0.001
Married	1008 (13.37)	508 (50.40)	500 (49.60)	
Unmarried	5993 (79.50)	3298 (55.03)	2695 (44.97)	
Widowed or divorced	537 (7.12)	240 (44.69)	297 (55.31)	
**Ethnicity**				0.586
Han	6773 (89.85)	3643 (53.79)	3130 (46.21)	
Minorities	765 (10.15)	403 (52.68)	362 (47.32)	
**Residence**				0.001
Eastern region	3351 (44.45)	1724 (51.45)	1627 (48.55)	
Western region	2640 (35.02)	1440 (54.55)	1200 (45.45)	
Central region	1547 (20.52)	882 (57.01)	665 (42.99)	
**Sexual orientation**				0.123
MSM	5695 (75.55)	3086 (54.19)	2609 (45.81)	
Bisexual or unsure	1843 (24.45)	960 (52.09)	883 (47.91)	
**Sexual risky behavior**				<0.001
Low	6003 (79.64)	3282 (54.67)	2721 (45.33)	
Moderate	1469 (19.49)	742 (50.51)	727 (49.49)	
High	66 (0.88)	22 (33.33)	44 (66.67)	
**Mpox-related knowledge**				<0.001
Low	537 (7.12)	172 (32.03)	365 (67.97)	
Moderate	636 (8.44)	296 (46.54)	340 (53.46)	
High	6365 (84.44)	3578 (56.21)	2787 (43.79)	
**Mpox virus infection**				0.014
No	7483 (99.27)	4026 (53.80)	3457 (46.20)	
Yes	55 (0.73)	20 (36.36)	35 (63.64)	
**Sexual diseases scores**				0.078
0	4798 (63.65)	2622 (54.65)	2176 (45.35)	
1~3	2688 (35.66)	1398 (52.01)	1290 (47.99)	
4~5	52 (0.69)	26 (50.00)	26 (50.00)	

Table S4. Influencing factors of self-funded mpox vaccination hesitation among men who have sex with men in China.

Characteristics	aOR (95% CI)	P-value
**Age group (years)**		
18-35	1 (reference)	
36-59	1.13 (1.00, 1.27)	0.054
≥60	1.47 (1.00, 2.16)	0.049
**Occupation**		
Employed	1 (reference)	
Unemployed	1.59 (1.36, 1.85)	<0.001
**Education level**		
Junior high school and below	1 (reference)	
High school	1.01 (0.85, 1.21)	0.918
Undergraduate	0.86 (0.72, 1.03)	0.097
Postgraduate and above	0.72 (0.58, 0.90)	0.004
**Marital status**		
Married	1 (reference)	
Unmarried	0.96 (0.83, 1.11)	0.588
Widowed or divorced	1.20 (0.96, 1.48)	0.103
**Residence**		
Eastern region	1 (reference)	
Western region	0.88 (0.79, 0.97)	0.013
Central region	0.86 (0.76, 0.98)	0.022
**Sexual risky behavior**		
Low	1 (reference)	
Moderate	1.11 (0.99, 1.25)	0.083
High	2.01 (1.18, 3.43)	0.010
**Mpox-related knowledge**		
Low	1 (reference)	
Moderate	0.54 (0.42, 0.68)	<0.0001
High	0.39 (0.33, 0.48)	<0.0001
**Mpox virus infection**		
No	1 (reference)	
Yes	1.62 (0.92, 2.86)	0.095

Notes: OR=odds ratios; 95%CI=95% confidence intervals.

Table S5. Influencing factors of mpox vaccination hesitation among men who have sex with men living without HIV in China.

Characteristics	aOR (95% CI)	P-value
**Age group (years)**		
18-35	1 (reference)	
36-59	1.05 (0.90, 1.23)	0.537
≥60	1.56 (1.02, 2.37)	0.038
**Occupation**		
Employed	1 (reference)	
Unemployed	1.54 (1.27, 1.87)	<0.001
**Education level**		
Junior high school and below	1 (reference)	
High school	1.09 (0.86, 1.38)	0.496
Undergraduate	1.00 (0.79, 1.28)	0.971
Postgraduate and above	0.88 (0.66, 1.17)	0.375
**Marital status**		
Married	1 (reference)	
Unmarried	0.95 (0.79, 1.14)	0.579
Widowed or divorced	1.24 (0.95, 1.63)	0.117
**Residence**		
Eastern region	1 (reference)	
Western region	0.88 (0.78, 1.00)	0.050
Central region	0.84 (0.73, 0.98)	0.023
**Sexual risky behavior**		
Low	1 (reference)	
Moderate	1.03 (0.89, 1.19)	0.696
High	1.92 (0.87, 4.23)	0.105
**Mpox-related knowledge**		
Low	1 (reference)	
Moderate	0.54 (0.41, 0.72)	<0.0001
High	0.40 (0.32, 0.50)	<0.0001
**Mpox virus infection**		
No	1 (reference)	
Yes	2.94 (1.29, 6.71)	0.011

Notes: OR=odds ratios; 95%CI=95% confidence intervals.

Table S6. Influencing factors of mpox vaccination hesitation among men who have sex with men living with HIV in China.

Characteristics	aOR (95% CI)	P-value
**Age group (years)**		
18-35	1 (reference)	
36-59	1.29 (1.04, 1.60)	0.019
≥60	1.50 (0.47, 4.82)	0.493
**Occupation**		
Employed	1 (reference)	
Unemployed	1.73 (1.33, 2.26)	<0.0001
**Education level**		
Junior high school and below	1 (reference)	
High school	0.97 (0.72, 1.30)	0.834
Undergraduate	0.65 (0.48, 0.88)	0.005
Postgraduate and above	0.43 (0.28, 0.67)	<0.001
**Marital status**		
Married	1 (reference)	
Unmarried	0.91 (0.66, 1.25)	0.546
Widowed or divorced	0.96 (0.65, 1.43)	0.853
**Mpox-related knowledge**		
Low	1 (reference)	
Moderate	0.58 (0.32, 1.05)	0.071
High	0.41 (0.25, 0.67)	<0.001

Notes: OR=odds ratios; 95%CI=95% confidence intervals.
